# A mouse model of progressive lung fibrosis with cutaneous involvement induced by a combination of oropharyngeal and osmotic minipump bleomycin delivery

**DOI:** 10.1152/ajplung.00408.2023

**Published:** 2024-04-23

**Authors:** Andrea Grandi, Erica Ferrini, Matteo Zoboli, Davide Buseghin, Francesca Pennati, Zahra Khalajzeyqami, Roberta Ciccimarra, Gino Villetti, Franco Fabio Stellari

**Affiliations:** ^1^Chiesi Farmaceutici S.p.A., Corporate Pre-Clinical R&D, Parma, Italy; ^2^Department of Veterinary Science, University of Parma, Parma, Italy; ^3^Dipartimento di Elettronica, Informazione e Bioingegneria, Politecnico di Milano, Milan, Italy; ^4^ANTHEM (AdvaNced Technologies for Human-centrEd Medicine), Milan, Italy; ^5^Department of Veterinary Medical Sciences, University of Bologna, Bologna, Italy

**Keywords:** bleomycin, interstitial lung disease, micro-CT imaging, osmotic mini-pump, systemic sclerosis

## Abstract

Systemic sclerosis (SSc) with interstitial lung disease (SSc-ILD) lacks curative pharmacological treatments, thus necessitating effective animal models for candidate drug discovery. Existing bleomycin (BLM)-induced SSc-ILD mouse models feature spatially limited pulmonary fibrosis, spontaneously resolving after 28 days. Here, we present an alternative BLM administration approach in female C57BL/6 mice, combining oropharyngeal aspiration (OA) and subcutaneous mini-pump delivery (pump) of BLM to induce a sustained and more persistent fibrosis, while retaining stable skin fibrosis. A dose-finding study was performed with BLM administered as 10 µg (OA) +80 mg/kg (pump) (10 + 80), 10 + 100, and 15 + 100. Forty-two days after OA, micro-computed tomography (micro-CT) imaging and histomorphometric analyses showed that the 10 + 100 and 15 + 100 treatments induced significant alterations in lung micro-CT-derived readouts, Ashcroft score, and more severe fibrosis grades compared with saline controls. In addition, a marked reduction in hypodermal thickness was observed in the 15 + 100 group. A time-course characterization of the BLM 15 + 100 treatment at *days 28*, *35*, and *42*, including longitudinal micro-CT imaging, revealed progressing alterations in lung parameters. Lung histology highlighted a sustained fibrosis accompanied by a reduction in hypodermis thickness throughout the explored time-window, with a time-dependent increase in fibrotic biomarkers detected by immunofluorescence analysis. BLM-induced alterations were partly mitigated by Nintedanib treatment. Our optimized BLM delivery approach leads to extensive and persistent lung fibrotic lesions coupled with cutaneous fibrotic alterations: it thus represents a significant advance compared with current preclinical models of BLM-induced SSc-ILD.

**NEW & NOTEWORTHY** This study introduces an innovative approach to enhance the overall performance of the mouse bleomycin (BLM)-induced model for systemic sclerosis with interstitial lung disease (SSc-ILD). By combining oropharyngeal aspiration and subcutaneous mini-pump delivery of BLM, our improved model leads to sustained lung fibrosis and stable skin fibrosis in female C57BL/6 mice. The optimized 15 + 100 treatment results in extensive and persistent lung fibrotic lesions and thus represents a significant improvement over existing preclinical models of BLM-induced SSc-ILD.

## Graphical abstract

**Figure:**
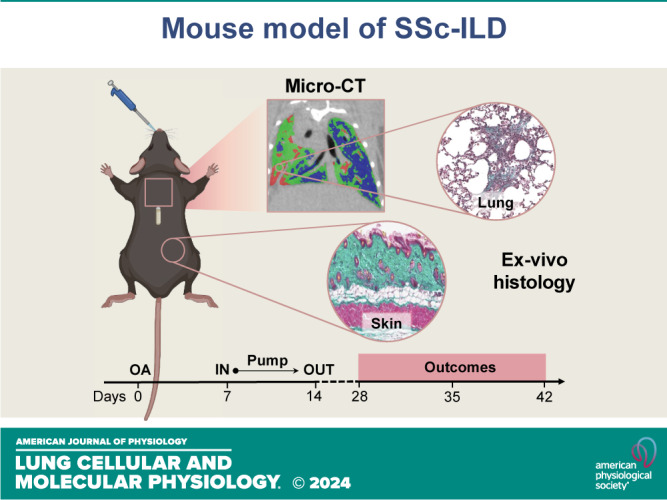

## INTRODUCTION

Progressive scleroderma or systemic sclerosis (SSc) is a rare systemic autoimmune and inflammatory disorder characterized by progressive fibrosis, immune dysregulation, and vasculopathy (1). SSc is frequently associated with interstitial lung disease (SSc-ILD), which dramatically worsens the life expectancy of patients (2). The etiology of SSc-ILD is still unknown. Treatment options are limited to symptomatic treatments, such as oxygen therapy, immunosuppressive therapies, and, in rapidly progressing and severe SSc, haematopoietic stem cell transplantation and lung transplantation (3, 4). Although two different drugs (i.e., the multikinase inhibitor Nintedanib and the anti-IL-6 antibody tocilizumab) have recently been approved for the treatment of SSc-ILD (5, 6), none of them can reverse disease progression. The lack of effective treatments thus represents a highly unmet medical need, whose amelioration strongly relies on animal model-based, preclinical studies.

Although there is presently no animal model capable of replicating all aspects of SSc-ILD, various experimental models have been investigated. One of these involves ectopic expression of the transcription factor Fra-2 (7) that induces multiorgan fibrosis but mainly affects pulmonary tissues in transgenic mice (8). However, the fibrosis-inducing agent most frequently used in rodents is the chemotherapeutic agent bleomycin (BLM), which exerts its profibrotic action through an incompletely elucidated mechanism involving oxidative stress, apoptosis, and inflammation (9). A variety of BLM-based animal models of lung fibrosis have been proposed in recent years, with special attention paid to the route, timing, and frequency of administration (10–12).

Continuous administration of BLM via subcutaneously implanted osmotic minipumps leads to multiorgan fibrosis, involving the skin, lungs, and other organs, resembling the systemic nature of SSc-ILD (11, 13). Compared with multiple subcutaneous injections, implanted osmotic minipumps allow a more standardized BLM administration while minimizing stress to the animals. Although this model ensures a stable and sustained skin fibrosis, as documented by alterations in dermis and hypodermis thickness, it causes only a mild form of lung fibrosis, primarily limited to the subpleural region. Moreover, the peak of lung fibrosis occurs at 21 days after pump implantation and tends to spontaneously resolve at subsequent time-points.

To bolster fibrosis induced by osmotic subcutaneous delivery (11, 14), we added an oropharyngeal aspiration (OA) of BLM (10, 12, 15) before mini-pump implantation to promote an enhanced, nonresolving pulmonary fibrosis, while retaining stable skin fibrosis.

To this end, female C57Bl/6 mice were first treated with different doses of OA-administered BLM, followed, 7 days later, by osmotic mini-pump implantation and subcutaneous delivery of varying amounts of BLM. In this model, based on a mixed mode of BLM administration we investigated the time-course of the fibrotic response by assessing lung and skin fibrotic alterations at different time-points. Micro-CT was used for longitudinal in vivo monitoring of pulmonary fibrosis progression in individual animals, whereas various histomorphometric analyses were performed to track both lung and skin lesions at each time-point. To further explore the relevance of the mixed-mode BLM administration model, a subgroup of animals was treated with Nintedanib daily for 2 wk, and treatment’s efficacy was assessed via micro-CT as well as histomorphometric analyses of lung and skin performed at the terminal time-point.

## MATERIALS AND METHODS

### Experimental Animals

Female inbred C57Bl/6j mice (8 wk old, 20 ± 1 g body wt) were purchased from Envigo, Italy (San Pietro al Natisone, Udine, Italy). Before use, animals were acclimatized for 7–10 days to the local vivarium conditions (room temperature: 20–24°C; relative humidity: 40–70%; 12-h light-dark cycle), with free access to regular rodent chow and softened tap water.

Animals were housed under standard conditions (5 mice/cage) at our animal facility, in compliance with the procedures and principles outlined in the European Directive 2010/63 UE, Italian D.Lgs 26/2014 and the revised “Guide for the Care and Use of Laboratory Animals” (National Research Council Committee, USA, 2011). All animal procedures were conducted in an AAALAC (Association for Assessment and Accreditation for Laboratory Animal Care) certified facility at Chiesi Farmaceutici and were authorized by the internal AWB (Animal Welfare Body) committee and by the Italian Ministry of Health with protocol number 841/2019-PR.

A Visual analogue scale (0–10) for pain assessment was assigned daily by a designated veterinarian or by trained technicians. VAS ≥6 and/or body weight loss ≥ 20% were considered as humane endpoints, as well as signs of dyspnoea or apathy. To prevent excessive body weight losses, animals’ diet was integrated with protein hydrogel, sterile sunflower seeds, and Meritene as recommended by the designated veterinarian.

### Study Protocols

**Figure 1.**
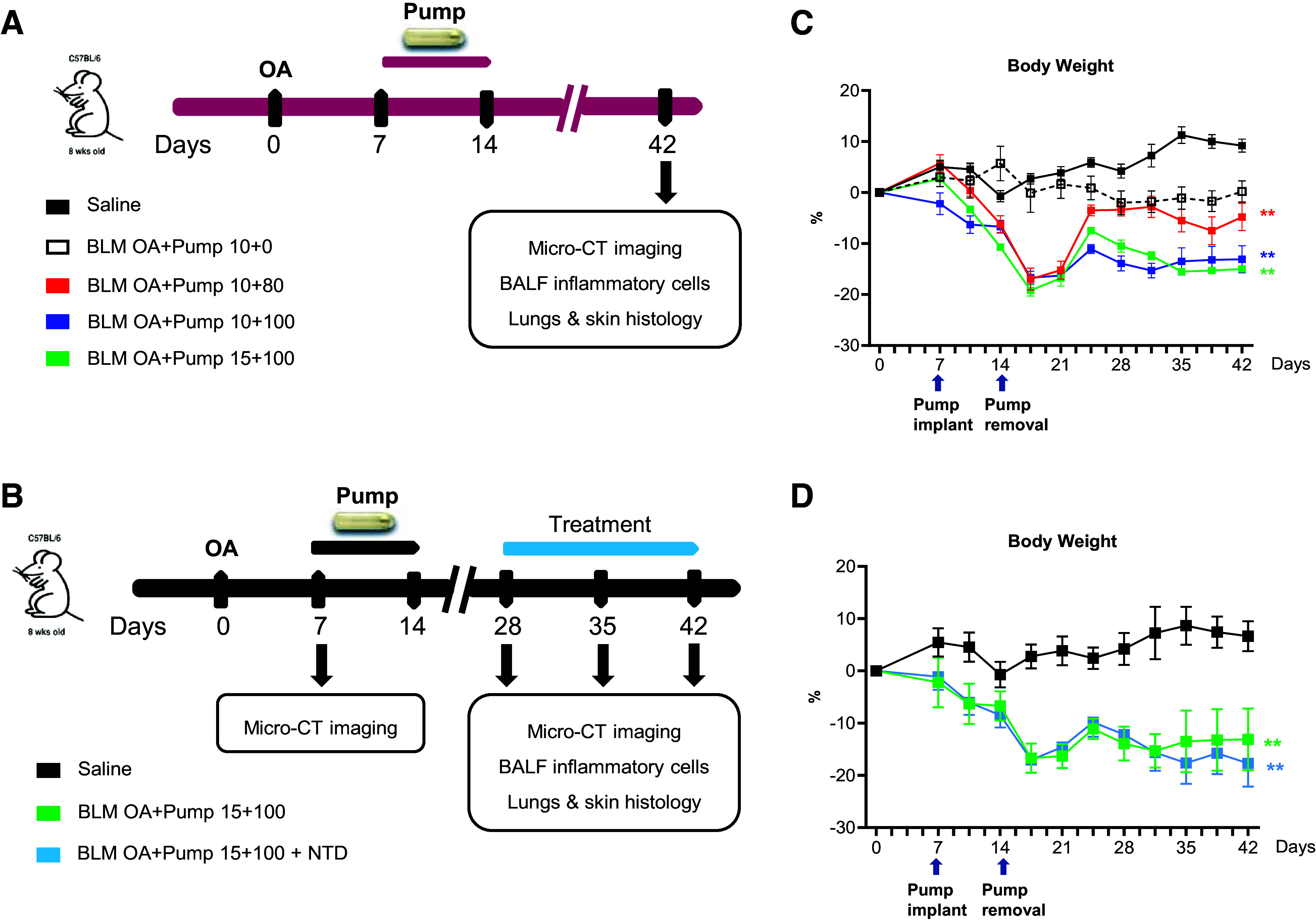
Schematic representation of the experimental setting and body weight variation over time. *A*: dose-finding study: 25 eight-wk-old female C57BL/6 mice were treated via OA with saline or bleomycin (BLM; 10 or 15 µg) at *day 0*; five animals were used for the saline and for each of the BLM OA+Pump groups. At *day 7*, osmotic mini-pumps filled with either saline or BLM (80 or 100 mg/kg) were implanted subcutaneously. At *day 14*, pumps were removed. At *day 42*, animals were subjected to noninvasive in vivo micro-CT imaging, BALF cells count, lung, and skin histology. *B*: time-course analysis and NTD treatment pilot study: 15 eight-weeks-old female C57BL/6 mice were treated via OA with saline or BLM (15 µg) at *day 0*, followed by micro-CT imaging at *day 7*. Osmotic mini-pumps filled with either saline or BLM (100 mg/kg) were then implanted subcutaneously and removed at *day 14* as above. At *days 28*, *35*, and *42* mice were examined longitudinally by noninvasive, in vivo micro-CT imaging. Subsets of 5 mice/group were euthanized at each time-point for BALF cells counting, plus lung and skin histology. An additional subset of five mice subjected to the BLM OA+Pump (15 + 100) mixed administration regimen were treated with NTD (60 mg/kg/day orally) from *day 28* to *day 42* and imaged by micro-CT only at *day 42* before euthanasia and ex vivo analysis. *C*: body weight variation in the saline and BLM OA+Pump (10 + 0, 10 + 80, 10 + 100, and 15 + 100) groups, given as mean ± SD for each time-point and for the various groups, expressed as percent variation compared with the body weight determined in the saline controls at *day 0*. Statistical significance was evaluated by two-way ANOVA with Tukey’s test for multiple comparisons; **P < 0.01 vs. saline. *D*: body weight variation in the saline, BLM OA+Pump (15 + 100), and BLM OA+Pump (15 + 100)+NTD treatment groups, expressed and statistically evaluated as specified in the legend to *B*. **P < 0.01 vs. saline. BALF, bronchoalveolar lavage fluid; micro-CT, micro-computed tomography; NTD, Nintedanib; OA, oropharyngeal aspiration.

The experimental procedure, with BLM OA administration at *day 0*, subcutaneous osmotic mini-pump implantation at *day 7* and removal at *day 14*, is outlined in Fig. 1, *A* and *B*.

In the first dose-finding study (Fig. 1*A*), mice were randomized into five different experimental groups (Table 1, *n* = 5 mice/group): the “saline control group” received the saline solution via OA and osmotic minipump; the ‘10 + 0’ group was given BLM via OA (10 µg/mouse) and then saline via osmotic minipump; the other three groups (‘10 + 80,’ ‘10 + 100,’ and ‘15 + 100’) were double-treated with different doses of BLM administered by OA (10 or 15 µg/mouse as indicated) and subcutaneous osmotic minipump (80, 100, or 150 mg/kg as indicated).

**Table 1.** Experimental groups utilized for the dose-finding study, n = 5 mice/group

Groups	Treatment
Saline control	50 µL OA + 100 µL Pump (Saline)
BLM OA+Pump 10 + 0	BLM 10 µg/mouse OA + 100 µL Saline
BLM OA+Pump 10 + 80	BLM 10 µg/mouse OA + 80 mg/kg Pump
BLM OA+Pump 10 + 100	BLM 10 µg/mouse OA + 100 mg/kg Pump
BLM OA+Pump 15 + 100	BLM 15 µg/mouse OA + 100 mg/kg Pump

BLM, bleomycin; OA, oropharyngeal aspiration.

Forty-two days after OA, animals were euthanized after micro-CT imaging. Following euthanasia, bronchoalveolar lavage fluid (BALF) was collected for the measurement of inflammatory cells, together with lung and skin samples to be used for histological analyses.

In a second longitudinal study (Fig. 1*B*), mice were given either saline or BLM via OA and pump at the highest BLM dosage, i.e., 15 µg/mouse of BLM via OA followed by 100 mg/kg BLM via sc osmotic minipump delivery (BLM OA + Pump 15 + 100). At *day 7*, i.e., before pump implantation, mice were subjected to micro-CT imaging, which was then repeated longitudinally on all animals at *days 28*, *35*, and *42* post-OA. At each time-point, subsets of five animals were euthanized and sampled for ex vivo analyses (i.e., BALF cells count, plus lung and skin histology). A subgroup of five BLM-treated mice received a daily oral gavage of Nintedanib (NTD, 60 mg/kg/day in a volume of 10 mL/kg, Carbosynth Limited, Compton, UK) dissolved in a saline solution of Tween 80 (0.05% vol/vol) from *day 28* to *day 42*.

### Oropharyngeal Aspiration

Mice received 50 µL of either saline (0.9% NaCl) or a solution containing 10 or 15 µg of BLM (Baxter) delivered by OA 7 days before pump implantation, which was considered as *day 0*.

Briefly, mice were slightly anesthetized with 2.5% isoflurane as described previously (10) placed on an intubation stand and, upon epiglottis visualization with a laryngoscope, the liquid was drained into the distal part of the oropharynx with a micropipette. Mice were then monitored until awakening and returned to their cages with free access to food and water.

### Bleomycin Delivery by Osmotic Mini Pumps

Twenty-four hours before implantation, osmotic mini pumps (ALZET1007D; DURECT Corporation, Cupertino, CA) were filled with 100 µL of either saline or BLM (16 or 20 mg/mL in saline, corresponding to 80 or 100 mg/kg, respectively) and activated under stirring conditions at 37°C overnight. The following day (*day 7*) each mouse was anesthetized with a 2.5% isoflurane-oxygen mixture. The anesthetic was delivered in an “anaesthesia box,” while for maintenance, mice were placed in prone position and a nose cone mask was used. The animals’ backs were shaved all around the implantation site, a small skin incision was made with surgical scissors, and a subcutaneous pocket was created using the jaws of a hemostat to position the pump. Osmotic minipumps were implanted under the back skin of the mice slightly caudal to the scapulae. The skin wound was cleaned with Betadine and closed with two surgical clips; after being returned to their cages, mice were monitored until full recovery. Osmotic mini pumps were set to deliver the liquid (saline or BLM solution) at 0.5 µL/h for 7 days.

Pumps were removed on the seventh day after implantation using the above-described anesthesia procedure. Mice were weighed daily throughout the experiment and the implantation scar was cleaned daily with Betadine until complete healing.

### Longitudinal Imaging by Micro-CT

Micro-computed tomography (micro-CT) lung imaging was performed longitudinally at *day 7*, *28*, *35*, and *42* using a Quantum GX Micro-CT (PerkinElmer Inc., Waltham, MA). Each mouse was anesthetized with 2% isoflurane and then positioned supine inside the CT scan box. Images were acquired with the following parameters: 90 KV, 88 μA over a total angle of 360° in 4-min-total scan time using the “high speed” scan mode with respiratory gating, to reconstruct the end-inspiration (P01) and end-expiration (P02) respiratory phases. The three-dimensional (3-D) reconstructed datasets were analyzed with the Analyze software (Analyze 12.0; Copyright 1986–2017, Biomedical Imaging Resource, Mayo Clinic, Rochester, MN). A semiautomatic segmentation procedure was used to define aerated lungs, whereas manual segmentation was performed in animals with very low- or nonaerated areas to obtain the total lung volume map (16). Images were rescaled from gray levels to Hounsfield units (HU) using a linear transformation model (−1,000 HU as air density and 0 HU as water density). The total number of voxels (N) and the mean lung attenuation (MLA) were computed for the segmented lungs at both respiratory phases, and several additional parameters were derived, as listed in Table 2.

**Table 2.** Functional and structural lung parameters derived from micro-CT scans^a^

	Name	Description	Unit	Formula
Micro-CT readouts (p = P01 or P02)	*N*_p_	Number of Voxel that compose segmentation	/	Number of voxels
	*V*_p_	Total Lung Volume	mm^3^	*N*_p_ × Voxel size
	MLA_p_	Mean Lung attenuation	HU	$\sum_{i=1}^{N_{p}}{\text{HU}}_{i}/N_{p}$
	Gas_p_	Content of gas inside the lung	mm^3^	$\frac{V_{p}\times {\text{MLA}}_{p}}{-1,000}$
Aeration compartments	%Normo	% Normo-aerated volume: % of lung with high content of gas	%	Percentage of voxel in HU range [−860; −435]
	%Hypo	% Hypo-aerated volume: % of lung with low content of gas	%	Percentage of voxel in HU range (−435; −121)
	%Non	% Non-aerated volume: % of lung with no gas	%	Percentage of voxel in HU range [−121; 121]
Micro-CT biomarkers of interest	Tissue	Volume of parenchyma without Gas component (calculated in P02)	mm^3^	*V*_P02_ – Gas_P02_
	%Gas_P01_	Percentage of gas inside the lung in P01	%	Gas_P01_ × 100/*V*_P01_
	%Gas_P02_	Percentage of gas inside the lung in P02	%	Gas_P02_ × 100/*V*_P02_

HU, Hounsfield units; micro-CT, micro-computed tomography. ^a^Parameters derived from micro-CT scans were divided into three main groups: *1*) micro-CT readouts of volumes and densities; *2*) aeration compartments, “normal,” “low,” and “no” gas, with the latter two considered as densitometric biomarkers of fibrosis; *3*) lung “tissue” and “gas” (%Gas_P01_ and %Gas_P02_) as structural biomarkers of inflammation and fibrosis.

### BALF Cells Count

Following euthanasia by anesthetic overdose and abdominal aorta excision, BALF was collected by gentle washing of the lungs (repeated three times) with 0.6 mL of sterile Hanks’ balanced salt solution (HBSS) [Hanks’ balanced salt solution (HBSS) 1×; ethylenediaminetetraacetic acid (EDTA) 10 mM; 4-(2-hydroxy-ethyl)-1-piperazineethanesulfonic acid (HEPES) 10 mM]. BALF samples were then centrifuged for 10 min (300 *g* at 4°C), the resulting cell pellet was resuspended in 0.2 mL of sterile HBSS solution and total white blood cells (WBC) as well as related WBC subpopulations (macrophages, lymphocytes, and neutrophils) were counted with an automated cell counter (Dasit XT 1800 J, Sysmex).

### Histological Analysis and Fibrosis Quantification

Following euthanasia, the whole lungs and a 1.5 × 1.5 cm skin fragments from the left gluteal region were collected. Both tissue samples were fixed in 10% neutral-buffered formalin for 24 h and histologically processed for paraffin embedding (Leica, HistoCore PEARLS). Three serial 5 μm thick sagittal sections stained with hematoxylin and eosin (H&E), Picrosirius red (PS), and Masson’s trichrome (MT) were used for histological analysis. Whole slide images (WSI) were acquired with a NanoZoomer S60 scanner (Hamamatsu Photonics K.K., Hamamatsu City, Japan).

Fibroproliferative modifications of the lungs were evaluated by a semiquantitative Ashcroft score (0 to 8) as modified by Hubner et al. (17–19). A frequency distribution of fibrotic alterations was generated by grouping and classifying lesions as mild (mean score from 0 to 3), moderate (4), and severe (5–8) (20).

Collagen content was determined on PS-stained sections using the QuPath v0.4.2 deep learning tool trained to detect collagen fibers by two independent operators (21).

For skin samples, the following histomorphometric parameters were evaluated on MT-stained sections: *1*) dermal thickness, defined as the mean distance between the epidermal-dermal junction (22); *2*) hypodermal thickness, defined as the mean distance between the dermal-subcutaneous junction and the muscle layer.

### Immunofluorescence Staining

Immunofluorescence analyses were performed on lung and skin sections (4 µm in thickness). Before immunofluorescence staining, paraffin-embedded sections were deparaffinized, rehydrated, and immersed in 10 mM citrate buffer at boiling temperature for antigen retrieval. After cooling, slides were rinsed in wash buffer and then incubated in blocking buffer (0.3 M glycine, 5% bovine serum albumin in 1× PBS; Sigma-Aldrich). For lung samples, primary antibodies anti-Col1a1 (5 µg/mL, ab88147; Abcam, Cambridge, UK) and anti-biglycan (10 µg/mL, bsm-54293R; Bioss, Zhuhai, PR China) were used to detect matrix components, whereas anti-CD68 (10 µg/mL, bsm1432R; Bioss, Zhuhai, PR China), anti-CD206 (1 µg/mL, AF2535; R&D System, Minneapolis), and anti-CD3 (1 µg/mL, ab5690; Abcam, Cambridge, UK) were utilized to detect monocytes, macrophages, and T cells, respectively. The same procedure, with anti-Col1a1, anti-CD68, and anti-CD206 primary antibodies, plus an antibody directed against the transcriptional regulator of macrophage differentiation peroxisome proliferator-activated receptor γ (PPAR-γ) (3 µg/mL, CPA4778; Cohesion Biosciences, London, UK) was used for skin samples. Secondary antibodies [AffiniPure goat anti-mouse IgG, subclass 3 specific Alexa Fluor-647 conjugated—(DIL 1:500) 111-545-144; AffiniPure Donkey anti-rabbit IgG (H + L) Rhodamine Red-X conjugated—(DIL 1:200) 711-295-152; AffiniPure Donkey anti-goat IgG (H + L) Alexa Fluor-488 conjugated—(DIL 1:500) 705-545-147, Jackson ImmunoResearch, Cambridge, UK]. Finally, DAPI (Cat. No. D1306, Invitrogen, 300 nM) was then added and incubated for 45 min at room temperature. A DAPI counterstain was used to mark cell nuclei and glass slides were mounted with ProLong Diamond Antifade Mountant (Cat. No. S36963, Invitrogen). Fluorescent whole slide images were then acquired with a Nanozoomer S60 slide scanner (Hamamatsu Photonics K.K., Hamamatsu City, Japan) equipped with specific filter wavelengths.

The Col1a1- and Biglycan-positive areas were quantified, normalized with respect to the whole tissue area for each sample, and expressed as percentage occupied area. Cellular components were examined following cell segmentation using DAPI counterstaining as a reference.

Within individual (sub)cellular areas, specific immunofluorescence signals were used to semiquantitatively detect monocytes (CD68^+^), macrophages M2-like (CD206^+^), monocytes-macrophages (CD68^+^-CD206^+^), lymphocytes T (CD3^+^), and peroxisome proliferator-activated receptor γ (PPAR-γ) positive cells; results were expressed as fold-increase with respect to saline.

### Statistical Analysis

Statistical analyses were conducted using Prism 8 software (GraphPad Software Inc., San Diego, CA). The Shapiro–Wilk test was used to check the normality assumptions of the data, supplemented by a visual examination of QQ-plots. Sample size determination was carried out through A-priori Power Analysis (GPower Version 3.1.2), utilizing the Ashcroft score as the end point.

Data are presented as means ± SD, as they are normally distributed. Initial analyses involved either one or two-way analysis of variance (ANOVA), followed by Dunnett, Sidak, or Tukey’s multiple comparison post hoc tests to enable comparisons among distinct experimental groups.

Significance was determined by a *P* value <0.05.

## RESULTS

### BLM Dose-Finding Study

#### Body weight assessment.

The results of body weight assessments comparing the four BLM dose regimen groups (BLM OA + Pump 10 + 0, 10 + 80, 10 + 100, and 15 + 100) to saline controls are reported in Fig. 1*C.* The 10 + 0 group displayed a nearly constant body weight throughout the entire study time-window (i.e., from *day 0* to *day 42*). The 10 + 80 group suffered a significant loss of body weight (*P* < 0.01 vs. saline), with a maximum drop around *days 17–20*, followed by recovery to a nearly normal body weight. Only a partial recovery was observed in the other two groups (10 + 100 and 15 + 100), whose body weights remained significantly below those of the saline controls till *day 42* (*P* < 0.01 vs. saline). In the longitudinal study (Fig. 1*D*), the BLM 15 + 100 group experienced a significant body weight loss (*P* < 0.01) compared with the saline controls, which was not significantly affected by the NTD treatment, except for a slight further drop between *days 35* and *42*.

#### Micro-CT results.

**Figure 2.**
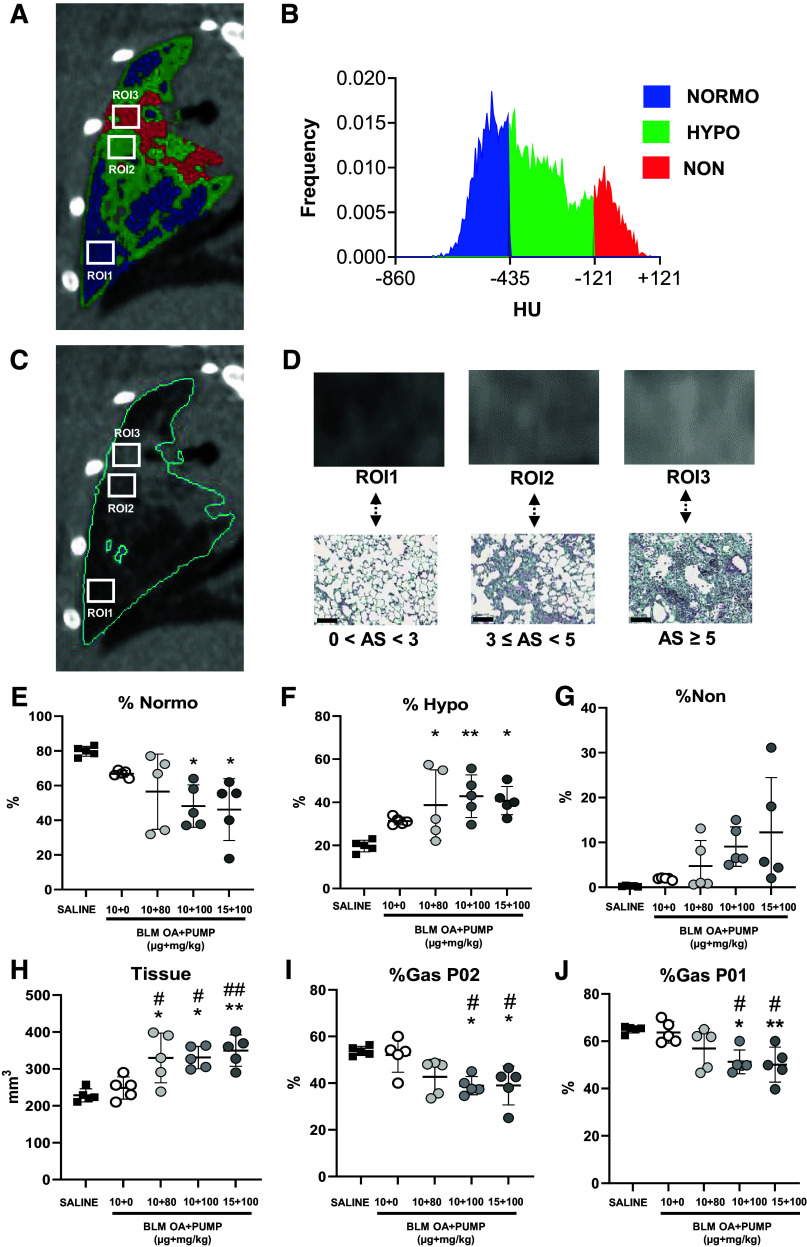
Dose-finding study: micro-CT analysis. *A–D*: explanatory description of the aeration compartments (i.e., Normo, Hypo, and Non) and their radiological features. Representative 2-D coronal micro-CT slide of the left lobe from a BLM-treated mouse, with the segmentation mask of densitometric compartments applied (*A*: Normo, blue; Hypo, green; Non, red) and the corresponding histogram with the Hounsfield units (HU) frequency distribution (*B*: Min = −860 HU; Max = +121; bins = 163). On the same 2-D coronal slide in gray scale (*C*), three rectangular ROIs (area = 0.8 mm^2^) with increasing fibrosis severity have been selected and reported at a ×4.4 magnification together with the corresponding histological Masson’s trichrome staining (*D*; scale bar: 100 µm). Micro-CT-derived parameters for the saline and the BLM OA+PUMP (10 + 0, 10 + 80, 10 + 100, and 15 + 100) groups determined at *day 42*, presented as scatter plots: %Normo (*E*), %Hypo (*F*), %Non (*G*), Tissue (*H*), %Gas_P02_ (*I*), and %Gas_P01_ (*J*) (see the text for details on the various parameters). Data were analyzed for statistical significance by one-way ANOVA+Tukey’s multiple comparison (*n* = 5 mice/group) and expressed as mean ± SD; *P < 0.05, **P < 0.01 vs. saline, #*P* < 0.05, ##*P* < 0.01 vs. BLM OA+Pump (10 + 0). BLM, bleomycin; micro-CT, micro-computed tomography; 2-D, two-dimensional.

A schematic representation of the different densitometric compartments (Normo, Hypo, and Non) revealed by micro-CT analysis and the radiological features observed in BLM-treated lungs are shown in Fig. 2, *A*–*D*. A two-dimensional (2-D) coronal slide of the left lobe is displayed as a representative example in Fig. 2, *A* and *C*. The HU frequency distribution of the selected 2-D lung slide is reported in Fig. 2*B*, with the HU ranges corresponding to Normo, Hypo, and Non colored in blue, green, and red, respectively. In Fig. 2*D*, the three rectangular regions of interest (ROIs, i.e., ROI1, ROI2, and ROI3; ∼ 0.8 mm^2^ each) selected and shown in Fig. 2, *A* and *C*, are reported with the corresponding Masson’s trichrome histological magnification. The gray color intensities of the three ROIs increase with increasing severity and turn from dark gray (ROI1) to light gray (ROI3) in a tissue density-dependent manner. The three different radiological patterns correlate with the reported histological patches and Ashcroft score ranges; also highlighted is the relative abundance of collagen deposited in the parenchyma as revealed by Masson’s trichrome staining.

The results of micro-CT analysis at *day 42* are shown in Fig. 2, *E*–*J*. Densitometric biomarkers (%Normo, %Hypo, and %Non) were non significantly altered in the BLM 10 + 0 group compared with the saline controls, whereas significant modifications were present in the other BLM OA + Pump groups. In particular, %Normo (Fig. 2*E*) was significantly decreased in the 10 + 100 and 15 + 100 groups (*P* < 0.05 vs. saline), and the %Hypo (Fig. 2*F*) was significantly increased in the 10 + 80 (*P* < 0.05 vs. saline), 10 + 100 (*P* < 0.01 vs. saline), and 15 + 100 groups (*P* < 0.05 vs. saline). A dose-dependent, albeit not statistically significant increase of %Non (Fig. 2*G*) was observed, with high variability within the groups. Among structural micro-CT parameters, “tissue,” reflecting extracellular matrix (ECM) deposition and inflammatory cells’ infiltration, was significantly increased in the 10 + 80, 10 + 100 (*P* < 0.05 vs. saline), and 15 + 100 (*P* < 0.01 vs. saline) groups, and with the same significance differed from that of the 10 + 0 group (Fig. 2*H*). %Gas was significantly lower in the 10 + 100 and 15 + 100 groups compared with the saline controls in the expiratory (P02; Fig. 2*I*, *P* < 0.05 for both groups) and inspiratory (P01; Fig. 2*J*; 10 + 100, *P* < 0.05; 15 + 100, *P* < 0.01) phases, whereas both groups significantly differed from the 10 + 0 group in both phases (*P* < 0.05).

#### Histomorphological results.

**Figure 3.**
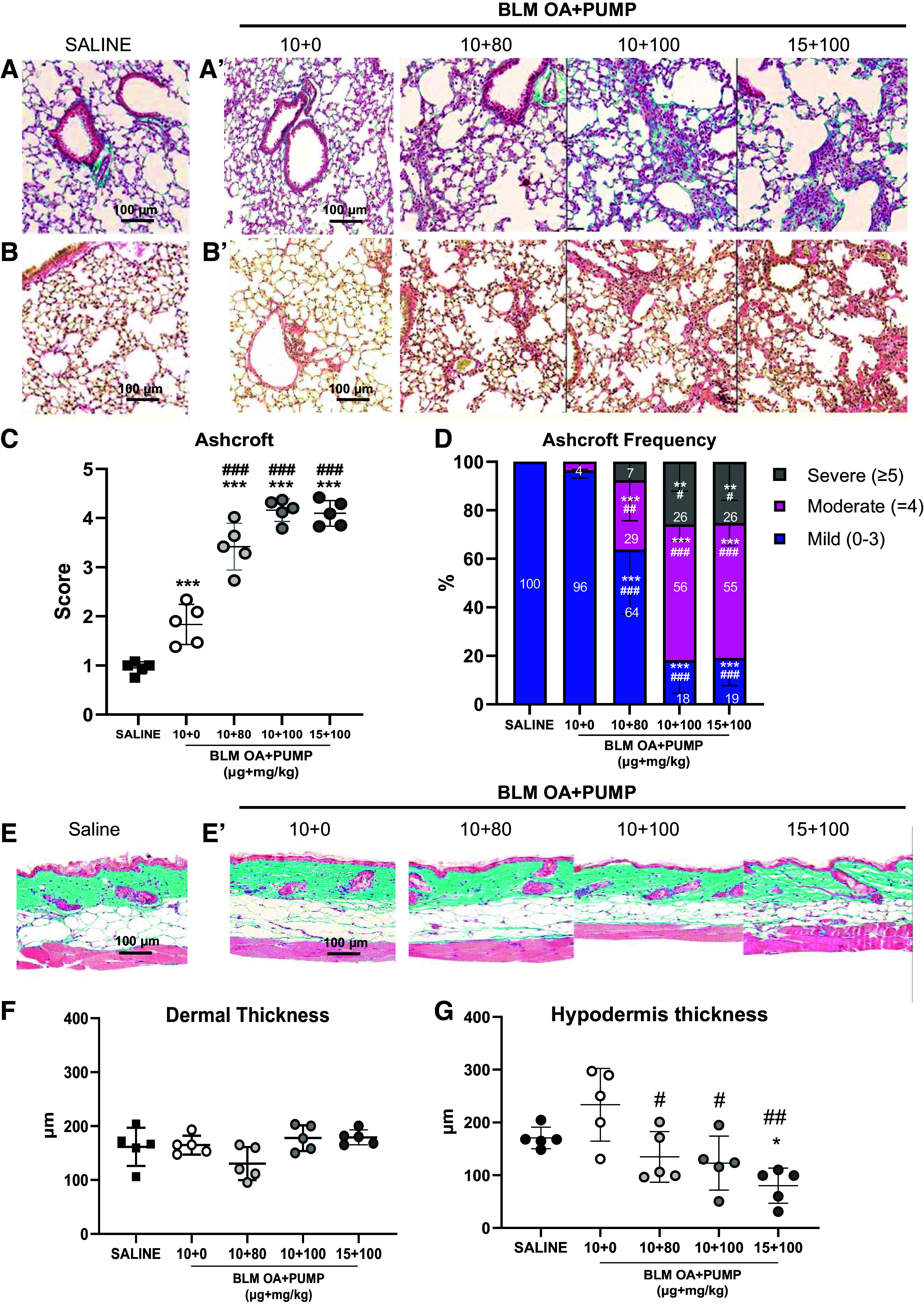
Dose-finding study: histological evaluation of lung parenchymal fibrosis and associated skin alterations. Lung sections (×20 magnification; scale bar: 100 µm) from saline controls and BLM OA+PUMP mice treated with different BLM dosages (10 + 0, 10 + 80, 10 + 100, and 15 + 100) were stained at *day 42* with MT (*A* and *A*’, respectively) and PS (*B* and *B*’, respectively). *C*: scatter plot representation of the Ashcroft scores given as mean ± SD for each experimental group. *D*: Ashcroft score frequency distribution expressed as fractional (%) representation (mean ± SD) of different fibrosis severity groups classified as mild (0–3), moderate (=4), and severe (≥5). MT-stained skin sections (×20 magnification; scale bar: 100 µm) from saline controls and BLM-treated mice at *day 42* (*E* and *E*’, respectively). Alterations in dermal (*F*) and hypodermal (*G*) thickness expressed as mean ± SD. For the data shown in *C*, *F*, and *G*, statistical significance was evaluated by one-way ANOVA+Tukey’s multiple comparison (*P < 0.05, ***P < 0.001 vs. saline; #*P* < 0.05, ##*P* < 0.01, ###*P* < 0.001 vs. BLM 10 + 0), whereas for the data in *D*, statistical significance was evaluated by two-way ANOVA+Tukey’s multiple comparison (**P < 0.01, ***P < 0.001 vs. saline; #*P* < 0.05, ##*P* < 0.01, ###*P* < 0.001 vs. BLM 10 + 0). BLM, bleomycin; micro-CT, micro-computed tomography; MT, Masson’s trichrome; OA, oropharyngeal aspiration; PS, Picrosirius red.

As documented by the tissue micrographs shown in Fig. 3, BLM induced a heterogeneous deposition of extracellular matrix in the lung. This appeared as a patchy accumulation of extracellular matrix components with varying degrees of confluence, which was particularly pronounced in MT- and PS-stained sections from BLM-treated (Fig. 3, *A*’ and *B*’) but not control (Fig. 3, *A* and *B*) mice.

Compared with the highest doses of the OA + Pump BLM treatment (i.e., 10 + 100 and 15 + 100), the lowest doses of BLM (10 + 80) as well the sole OA administration of bleomycin (BLM 10 + 0) led to much milder fibrotic lesions. These findings align with the results obtained in vivo by micro-CT imaging.

The severity of fibrosis on lung parenchyma quantified by the Ashcroft score (Fig. 3*C*) was significantly increased with respect to saline in all the BLM-treated groups, with a progressive score increase with increased BLM doses, particularly with the OA + Pump, 10 + 100, and 15 + 100 BLM treatments (*P* < 0.001). Moreover, mixed-mode BLM administration induced a significant increase in the Ashcroft score for these three groups (i.e., 10 + 80, 10 + 100, and 15 + 100) compared with the BLM OA + Pump 10 + 0 group (*P* < 0.001). The Ashcroft score frequency distribution (Fig. 3*D*), in the OA + Pump BLM 10 + 80 treatment showed a significant increase in moderate-degree fibrosis (*P* < 0.001 vs. saline; *P* < 0.01 vs. OA + Pump, 10 + 0 BLM) and a reduction of mild-degree fibrosis (*P* < 0.001). However, only the highest doses, 10 + 100 and 15 + 100, caused a significant increase in severe-degree fibrosis (*P* < 0.01 vs. saline; *P* < 0.05 vs. 10 + 0 BLM), along with a raise in moderate-degree fibrosis (*P* < 0.001 vs. saline and 10 + 0 BLM).

As to histomorphologically detectable skin alterations, only mice treated with the highest BLM doses (OA + Pump 10 + 100 and 15 + 100) displayed appreciable skin modifications, highlighted by an increased collagen compactness in the dermal area and a reduced hypodermal layer (Fig. 3, *E* and *E*’).

Although dermal thickness remained almost unchanged following 10 + 0 and 10 + 80 BLM treatments (Fig. 3*F*), a moderate, albeit nonsignificant increase was observed in the BLM 10 + 100 and 15 + 100 groups. Moreover, a dose-dependent reduction in hypodermis thickness was observed in the BLM OA + Pump-treated mice and reached statistical significance compared with the saline controls for the 15 + 100 group (*P* < 0.05 vs. saline; Fig. 3*G*), whereas all doses significantly differed from the BLM OA + Pump 10 + 0 group (10 + 80 and 10 + 100, *P* < 0.05; 15 + 100, *P* < 0.01).

Regarding BALF cells, WBC levels were not significantly modified in the BLM 10 + 0 group, despite a moderate increase compared with the saline controls (Supplemental Fig. S1*A*). WBCs significantly increased in all the other BLM OA + Pump groups (10 + 80 *P* < 0.05 vs. saline; 10 + 100 *P* < 0.01 vs. saline), with the largest increase observed in the 15 + 100 group (*P* < 0.01 vs. saline). The WBC increase measured with the highest dose was also found to be significantly higher compared with the BLM OA + Pump 10 + 0 group (*P* < 0.05; Supplemental Fig. S1*A*). Similarly, as shown in Supplemental Fig. S1*B*, macrophage (MP) levels were found to be dose dependently higher compared with saline controls in all BLM OA + Pump groups (10 + 80 *P* < 0.05 vs. saline; 10 + 100 and 15 + 100 *P* < 0.01 vs. saline). In particular, they were significantly higher in the 15 + 100 group compared with the 10 + 0 group (*P* < 0.01). Lymphocyte levels were similarly found to be considerably increased in all BLM OA + Pump groups compared with saline controls (*P* < 0.05 vs. saline for the 10 + 80 and 10 + 100 groups), with the most pronounced increment observed at the highest dosage (15 + 100 *P* < 0.01 vs. saline), which also turned out to be significantly higher than the BLM 10 + 0 dosage (*P* < 0.01; Supplemental Fig. S1*C*). No significant variation in neutrophil levels was observed in any of the different treatment groups (Supplemental Fig. S1*D*).

### Longitudinal Micro-CT Evaluation of Lung Fibrosis Progression Induced by Mixed-Mode Treatment with the Highest BLM Dosage (15 + 100)

**Figure 4.**
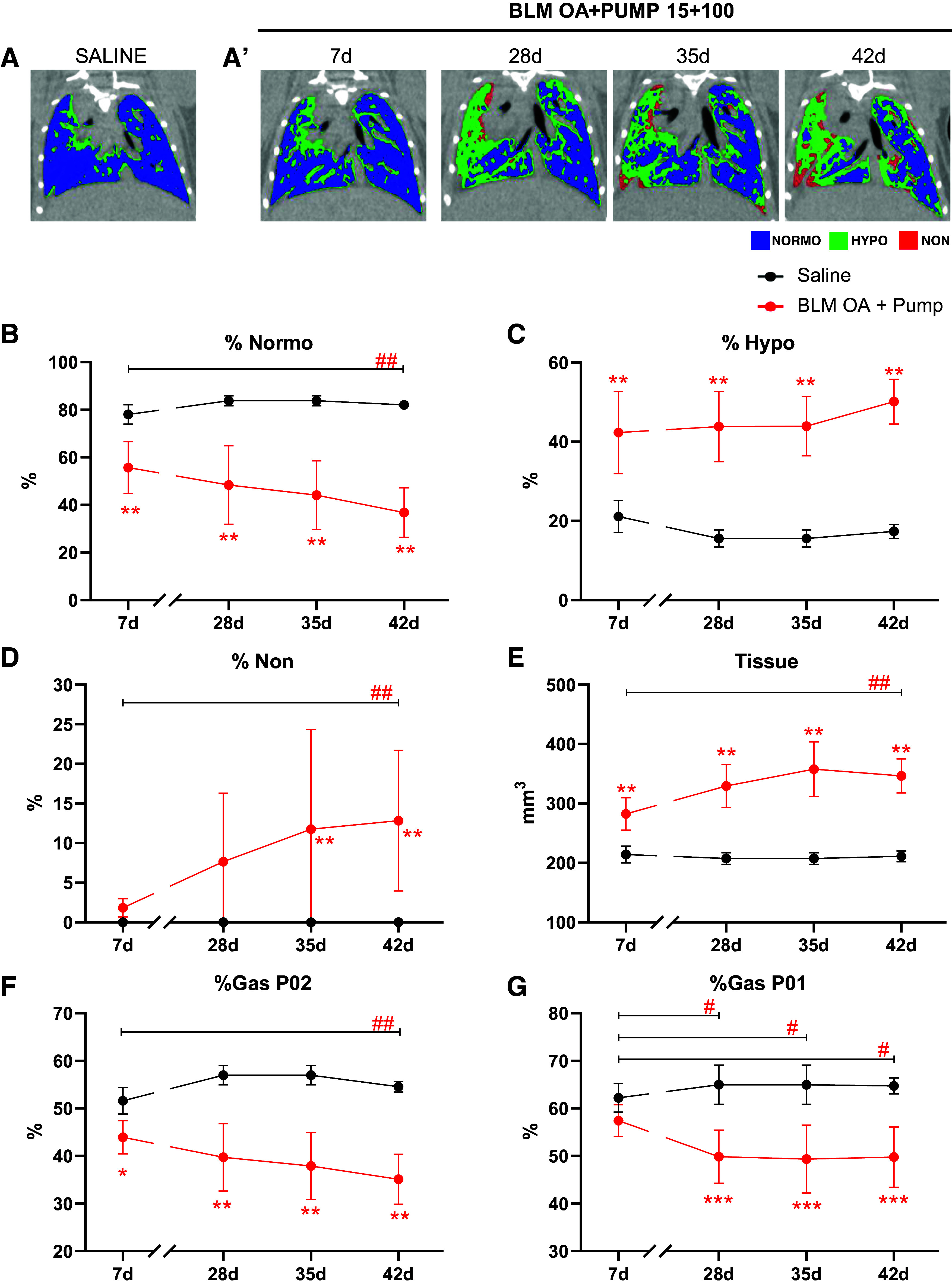
Time-course experiment: longitudinal assessment of lung fibrosis by micro-CT imaging. Representative coronal micro-CT lung slides at different time points (7, 28, 35, and 42 days post-OA), recorded at the end of the expiration phase in saline controls (*A*) and BLM OA+PUMP 15 + 100-treated mice (*A*’). Normo-aerated, hypo-aerated, and non-aerated compartments are rendered in blue, green, and red, respectively. Lung aeration compartments, %Normo (*B*), %Hypo (*C*), %Non (*D*), and other micro-CT-derived biomarkers: Tissue (mm^3^) (*E*), %Gas_P02_ (*F*), and %Gas_P01_ (*G*), determined in saline controls and in BLM OA+PUMP (15 + 100)-treated mice at *day 7* (*n* = 15 mice/group), 28 (*n* = 15 mice/group), 35 (*n* = 10 mice/group), and 42 (*n* = 5 mice/group) post-OA. Data, given as mean ± SD values for the various groups, and time-points, are expressed as the fractional representation of differently aerated lung areas (%). “Tissue” data are expressed as mm^3^. Statistical significance was evaluated by two-way ANOVA with Tukey’s test for multiple comparisons; *P < 0.05, **P < 0.01, ***P < 0.001 vs. saline; #*P* < 0.05, ##*P* < 0.01 vs. *day 7*. BLM, bleomycin; micro-CT, micro-computed tomography; OA, oropharyngeal aspiration.

Representative coronal micro-CT images for the saline and the BLM (OA + Pump) groups are reported in Fig. 4, *A* and *A*’, respectively, which show a decrease in blue (Normo) in favor of the green (Hypo) and red (Non) regions. As illustrated in Fig. 4*B*, the BLM-treated group featured a significantly lower %Normo value compared with the saline group at *day 7* (i.e., immediately before pump implantation; *P* < 0.01 vs. saline), which gradually decreased thereafter (i.e., from *day 28* to *day 42*; *P* < 0.01 vs. saline). In keeping with this trend, %Hypo was significantly higher in the BLM-treated group compared with saline (*P* < 0.01): it remained essentially constant until *day 35*, showing a rapid increase from *day 35* to *42* (Fig. 4*C*). The %Non, which delineates the most severely damaged lung parenchyma, was significantly increased in the BLM-treated mice with respect to saline at *days 35* and *42* (*P* < 0.01; Fig. 4*D*). Tissue significantly increased in the mixed-mode BLM-treated animals than in saline controls at all the examined time-points (*P* < 0.01): it peaked at *day 35* and remained essentially constant up to *day 42* (Fig. 4*E*). The percentage of gas significantly decreased in BLM-treated mice both at end-expiration (P02) starting from *day 7* (*P* < 0.05, Fig. 4*F*) and at end-inspiration (P01) from *day 28* to *day 42* (*P* < 0.001, Fig. 4*G*).

### Histological Assessment of Fibrotic Lesions Induced by Mixed-Mode Treatment with the Highest BLM Dosage

**Figure 5.**
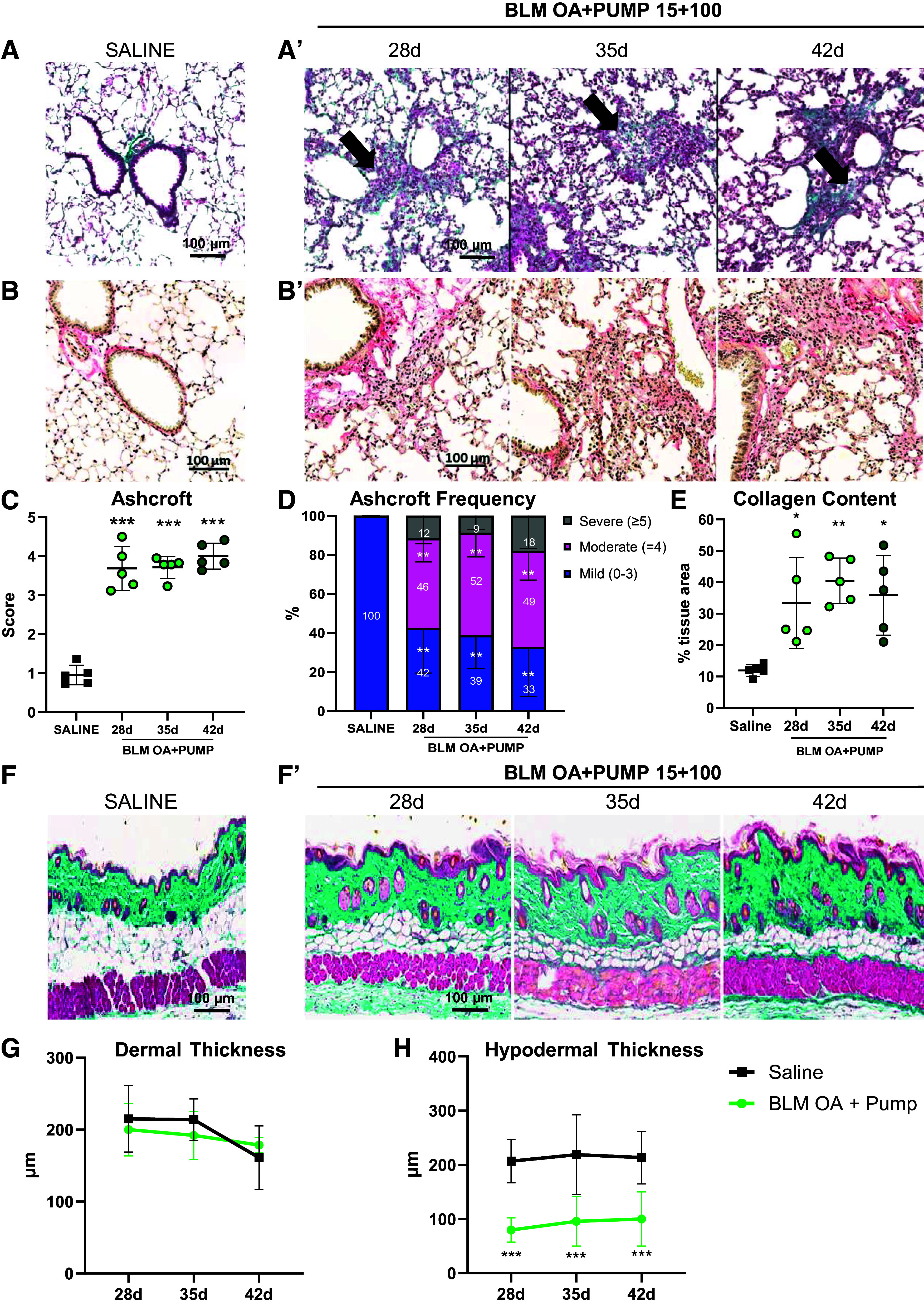
Time-course experiment: histomorphometric analysis of lung and skin fibrosis progression following bleomycin administration. Representative MT- and Picrosirius-stained lung sections (×20 magnification; scale bar: 100 µm) from saline controls and BLM OA+PUMP (15 + 100)-treated mice at different time-points (28, 35, and 42 days post-OA) are shown in *A–A*’ and *B–B*’, respectively. Ashcroft scores represented as scatter plots with mean ± SD for each time-point and treatment condition (saline and BLM OA+PUMP 15 + 100; *n* = 5 mice/group/time-point) are shown in *C*. Ashcroft scores frequency distribution of different severity groups, classified as mild (0–3), moderate (=4), and severe (≥5), is expressed as fractional (%) representation and plotted as mean ± SD in *D*. Collagen content quantification performed on Picrosirius sections is expressed as % tissue area of the whole slide area (mean ± SD; *n* = 5 mice/group/time-point) and presented as a scatter plot in *E*. Representative MT-stained skin sections (×20 magnification; scale bar: 100 µm) from saline controls and BLM-OA + PUMP (15 + 100)-treated mice at the indicated time-points after OA-BLM administration (28, 35, and 42 days) are shown in *F* and *F*’, respectively. Dermal thickness (*G*) and hypodermal thickness (*H*) variations in saline controls and BLM OA+PUMP (15 + 100)-treated mice at the indicated time-points (*n* = 5 mice/group/time-point); data (µm) are given as mean ± SD. For *C* and *E* statistical significance was evaluated by one-way ANOVA with Tukey’s test for multiple comparisons (*P < 0.05, **P < 0.01, ***P < 0.001 vs. saline), whereas for *D*, *G*, and *H*, two-way ANOVA with Tukey’s test for multiple comparisons was used (**P < 0.01, ***P < 0.001 vs. saline). BLM, bleomycin; MT, Masson’s trichrome; OA, oropharyngeal aspiration.

Lung parenchymal alterations induced by mixed-mode BLM administration resulted in well-detectable fibroproliferative changes at 28, 35, and 42 days compared with the saline controls (Fig. 5, *A*’ and *A*). Extracellular matrix deposition was assessed by collagen fiber quantification on PS-stained sections (Fig. 5, *B* and *B*’). In line with micro-CT data, the Ashcroft score significantly increased after BLM mixed mode (15 + 100) administration at all time-points (*P* < 0.001), with a particularly sustained fibrosis at *day 42* (Fig. 5*C*). Ashcroft frequency distribution similarly highlighted a significant increase of moderate-degree fibrosis in the BLM group at all the examined time-points (*P* < 0.01 vs. saline), with a parallel significant reduction of mild-degree fibrosis (*P* < 0.01 vs. saline; Fig. 5*D*). Fields indicative of severe fibrosis were observed all time-points after BLM mixed-mode (15 + 100) administration, with an appreciable, albeit statistically nonsignificant increase at *day 42* post-OA.

A statistically significant increase of the fractional area (%) occupied by collagen fibers (Fig. 5*E*) was observed in the BLM (15 + 100) treatment group compared with saline controls at *days 28* and *42* (*P* < 0.05), with a peak value at *day 35* (*P* < 0.01).

As to morphological skin alterations (see representative micrographs for saline controls and BLM-treated mice in Fig. 5, *F* and *F*’, respectively), a moderate, albeit nonsignificant decrease of dermal thickness was detected in BLM-treated mice throughout the entire experimental time-course (Fig. 5*G*). At variance with this fairly modest dermal thickness variation, a significant reduction of hypodermal thickness was consistently observed in BLM-treated mice at all time-points (*P* < 0.001 vs. saline; Fig. 5*H*).

### Immunofluorescence Analysis of the Lungs and Skin of Mice Subjected to Mixed-Mode BLM (15 + 100) Treatment

**Figure 6.**
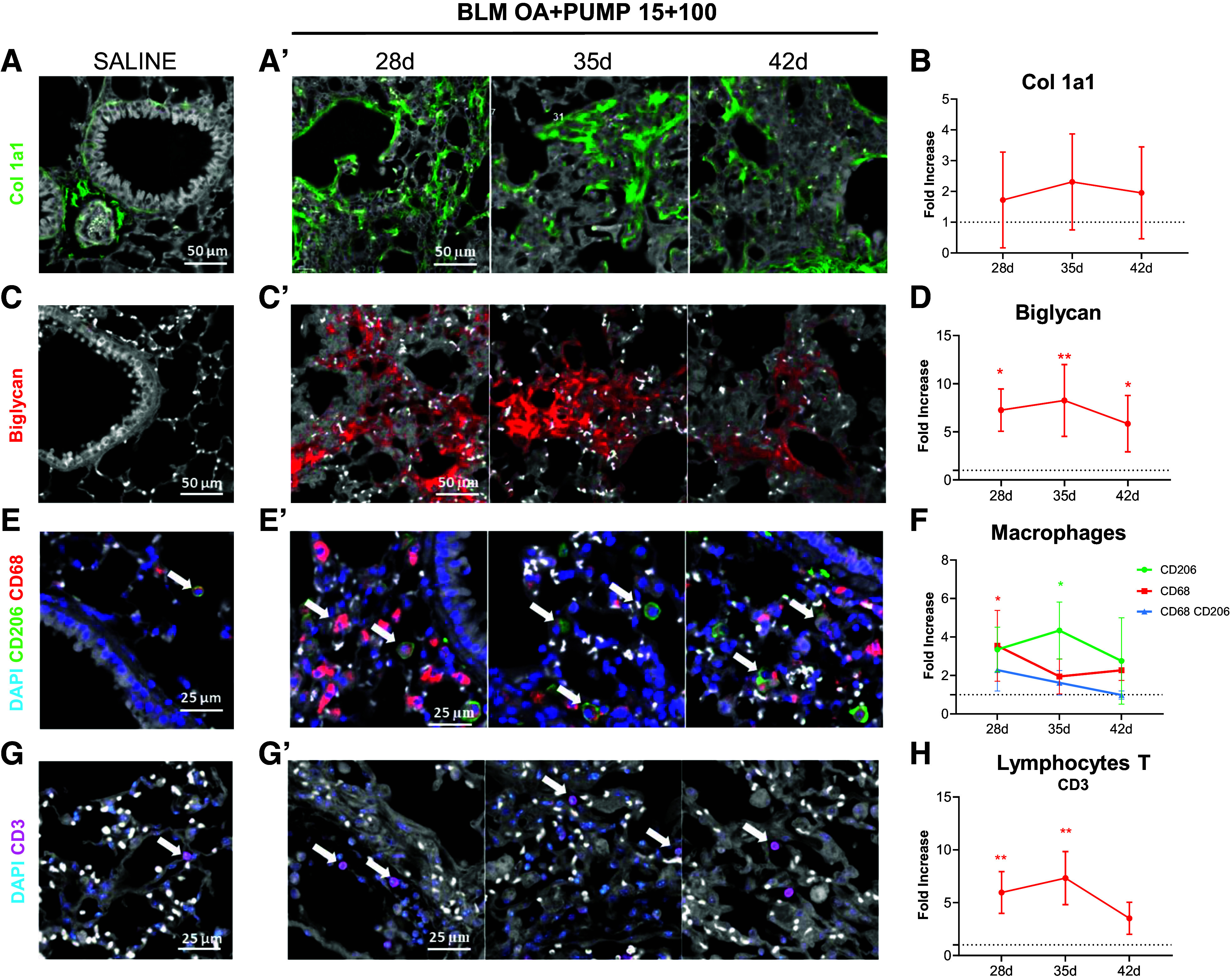
Extracellular matrix remodeling and inflammatory cells recruitments in the lung parenchyma of BLM-OA+PUMP-treated mice. Immunofluorescence (IF) analysis of lung parenchyma (×20 magnification; scale bar: 50 µm) from saline controls and BLM OA+PUMP (15 + 100)-treated mice at the indicated time-points (28, 35, and 42 days post-OA). IF staining for collagen type I (Col 1a1; shown in green in *A* and *A*’ for saline controls and BLM-treated mice, respectively) and Biglycan (shown in red in *C* and *C*’ for saline controls and BLM-treated mice, respectively). IF staining for CD68^+^ monocytes and M-2 like CD206^+^ macrophages (shown in red and green in *E* and *E*’) and CD3^+^ T cells (shown in red in *G* and *G*’ for saline controls and BLM-treated mice, respectively); the relevant WBCs (×40 magnification; scale bar: 25 µm) are marked with white arrows; nuclei are counterstained with DAPI (blue). IF data collected at each time point after BLM administration are quantified and expressed as fold-increase ± SD with respect to the saline controls in *B*, *D*, *F*, and *H*. Statistical significance was evaluated by one-way ANOVA with Tukey’s test for multiple comparisons (*P < 0.05, **P < 0.01 vs. saline). BLM, bleomycin; OA, oropharyngeal aspiration.

A more detailed investigation of the fibrotic and inflammatory status was performed by an immunofluorescence analysis of both the lungs and the skin using a suite of antimatrix components and anti-WBCs antibodies (see ‘materials and methods’ for details). Immunofluorescence data confirmed an increase in lung extracellular matrix components, collagen 1a1 (Fig. 6, *A*, *A*’, and *B*) and biglycan (Fig. 6, *C*, *C*’, and *D*), at all the examined time-points in mice subjected to mixed-mode BLM administration (15 + 100) compared with saline controls. More specifically, a statistically significant increase in biglycan (*P* < 0.01) was detected at *days 28*, *35*, and *42* (Fig. 6, *C*, *C*’, and *D*). Evaluation of the inflammatory cellular (WBC) components of the lung parenchyma also revealed an increase in monocytes and macrophages. In particular, a significant increase in monocytes/macrophages (CD68^+^) at *day 28* and a significant increase in M2-like macrophages (CD206^+^) at *day 35* (*P* < 0.05) was observed in BLM-treated animals (Fig. 6, *E*, *E*’, and *F*). Similarly, T-cell lymphocytes (CD3^+^) significantly increased at *days 28* and *35* (*P* < 0.01 vs. saline, Fig. 6, *G*, *G*’, and *H*).

**Figure 7.**
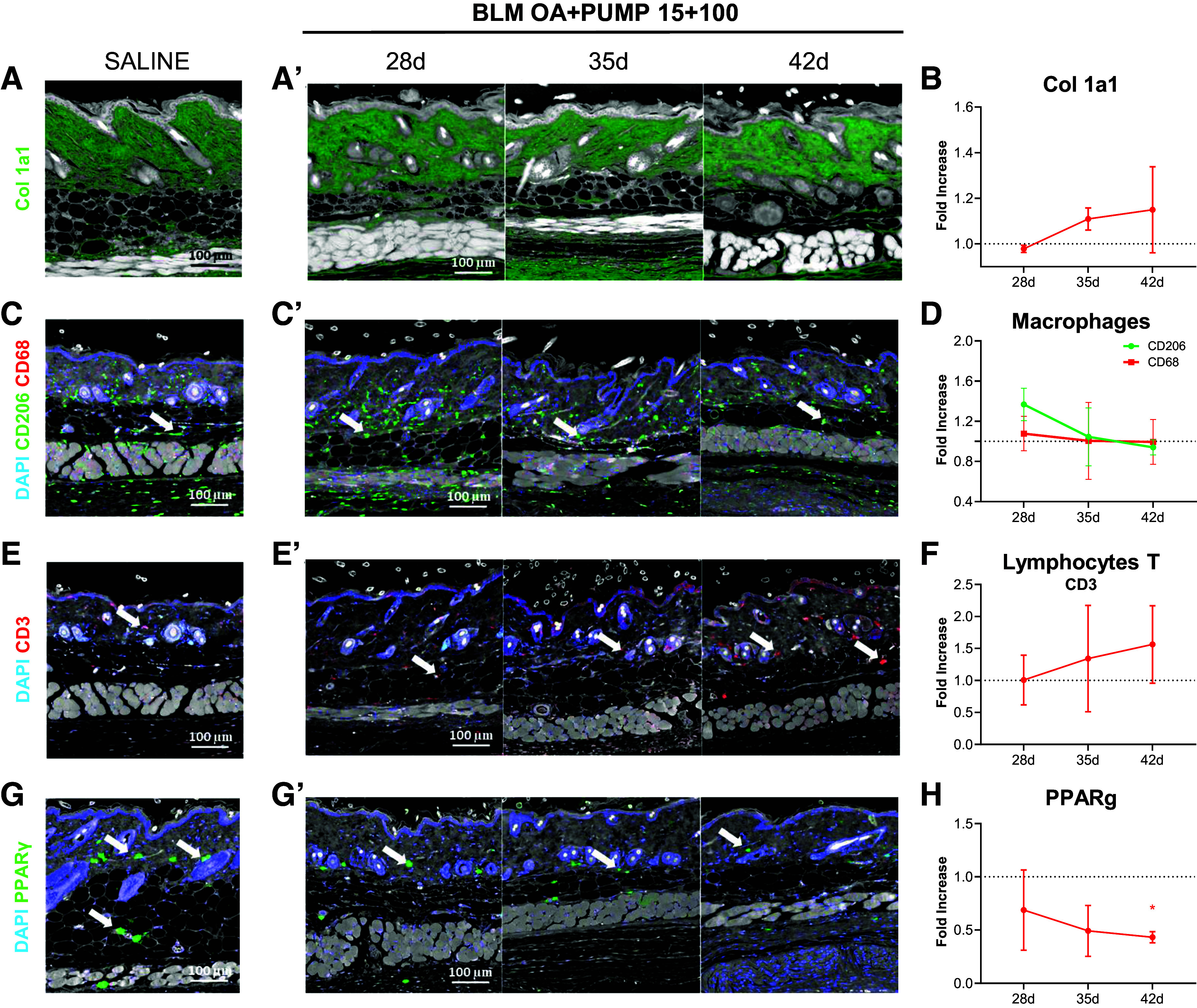
Skin alterations and inflammatory cell migration during progression of BLM-induced fibrosis. Immunofluorescence (IF) staining for collagen type I (*Col 1a1*) in saline and BLM OA+PUMP (15 + 100)-treated mice at 28, 35, and 42 days post-OA (shown in green in *A* and *A*’, respectively); ×10 magnification (scale bar: 100 µm). IF staining at the indicated time-points for CD68^+^ monocytes, M-2 like CD206^+^ macrophages (shown in red and green in *C* and *C*’) and CD3^+^ T cells (shown in red in *E* and *E*’ for saline controls and BLM-treated mice, respectively); the relevant WBCs (×10 magnification; scale bar: 100 µm) are marked with white arrows; nuclei are counterstained with DAPI (blue). PPAR-γ levels determined in saline controls and BLM-OA + PUMP (15 + 100)-treated mice at the indicated time-points (shown in green in *G* and *G*’, respectively). IF data collected and quantified at each time point after BLM administration are expressed as fold-increase ± SD with respect to the saline controls in *B*, *D*, *F*, and *H*. Statistical significance was evaluated by one-way ANOVA with Tukey’s test for multiple comparisons (*P < 0.05 vs. saline). BLM, bleomycin; OA, oropharyngeal aspiration; PPAR-γ, peroxisome proliferator-activated receptor γ.

Collagen 1a1 deposition in the skin progressively increased in BLM-treated animals compared with saline controls up to *day 42* (Fig. 7, *A*, *A*’, and *B*), whereas biglycan immunostaining in the skin resulted in a signal that was too low to be quantified (data not shown).

A slight but nonsignificant increase in M2-like macrophages was detected at *day 28* (Fig. 7, *C*, *C*’, and *D*). Similar increases were observed for Col 1a1 and T lymphocytes (CD3+) up to *day 42* (Fig. 7, *E*, *E*’, and *F*).

Finally, PPAR-γ, a transcriptional regulator whose activation primes the monocyte-macrophage (CD68^+^-CD206^+^) M2 phenotype, gradually declined in mixed-mode BLM-treated animals compared with saline controls (*P* < 0.05 on *day 42*; Fig. 7, *G*, *G*’, and *H*).

### BALF Cellular Components of Mice Subjected to Mixed-Mode BLM Treatment

The time-dependent distribution (28, 35, and 42 days) of different inflammatory cells in the BALFs of saline controls and mice subjected to mixed-mode BLM administration (15 + 100) is presented in Supplemental Fig. S1*E*. Total white blood cells (WBC) were significantly higher (*P* < 0.01) in BLM-treated (15 + 100) mice compared with saline controls at all the investigated time-points, with only a slight decrease between *days 35* and *42*. A similar trend was observed for the macrophage component, which significantly increased in BLM-treated mice (*P* < 0.01 vs. saline) at *days 28*, *35*, and *42*, albeit with a slight reduction at the latter time-point (*P* < 0.05 vs. saline). Lymphocyte levels also significantly increased in BLM-treated animals at *day 35* (*P* < 0.01 vs. saline). Neutrophil (PMN) levels, instead, were low and close to baseline throughout the entire time-course.

### Micro-CT and Histomorphometric Evaluation of Nintedanib Treatment

**Figure 8.**
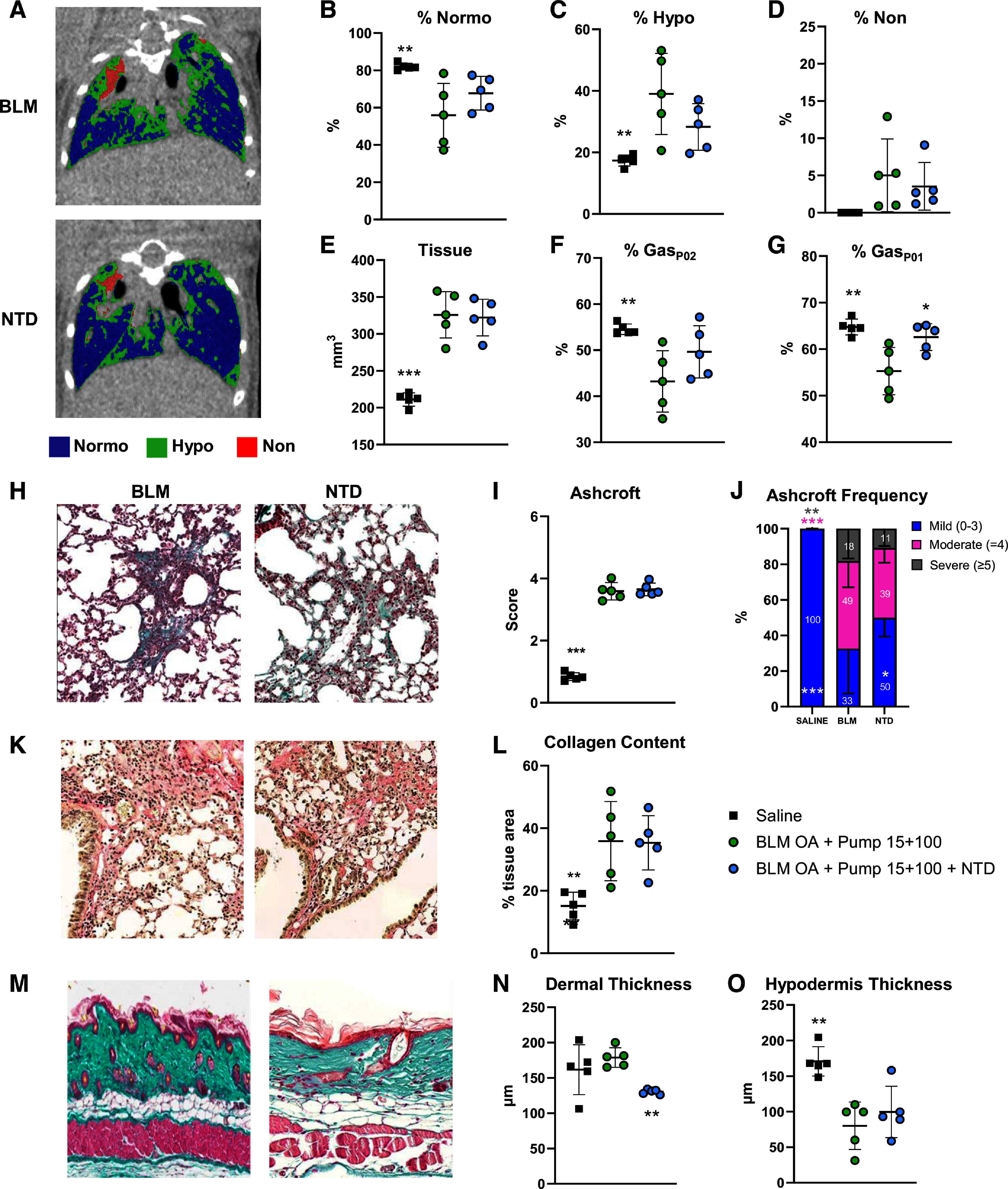
Micro-CT and histomorphometric assessment of lung and skin fibrosis in Nintedanib-treated mice. *A*: representative coronal micro-CT lung slides at *day 42* post-OA, recorded at the end of the expiratory phase in BLM OA+PUMP (15 + 100) and BLM OA+PUMP (15 + 100+NTD)-treated mice. Micro-CT-derived parameters (mean ± SD) determined on the saline, BLM OA+PUMP (15 + 100), and BLM OA+PUMP (15 + 100+NTD) groups at *day 42* post-OA, represented as scatter plots: %Normo (*B*), %Hypo (*C*), %Non (*D*), Tissue (*E*), %Gas_P02_ (*F*), and %Gas_P01_ (*G*) (see the text for details on the various parameters). Representative MT-stained lung sections (×20 magnification; scale bar: 100 µm) from BLM OA+PUMP (15 + 100) and BLM OA+PUMP (15 + 100+NTD) mice at *day 42* (*H*). Scatter plot representation of the Ashcroft scores (mean ± SD) for the saline, BLM OA+PUMP (15 + 100) and BLM OA+PUMP (15 + 100+NTD) groups (*n* = 5 mice/group) (*I*). Ashcroft score frequency distribution expressed as fractional (%) representation (mean ± SD) of different fibrosis severity groups classified as mild (0–3), moderate (=4), and severe (≥5); statistical significance was evaluated by two-way ANOVA+Dunnett’s multiple comparison (*P < 0.05, **P < 0.01, ***P < 0.001 vs. BLM OA+PUMP 15 + 100) (*J*). Representative PS-stained lung sections (×20 magnification; scale bar: 100 µm) from BLM OA+PUMP (15 + 100) and BLM OA+PUMP (15 + 100+NTD) mice at *day 42* (*K*). *L*: collagen content quantification (mean ± SD) derived from Picrosirius-stained sections, given as % tissue area of the whole slide area and presented as a scatter plot (*n* = 5 mice/group/time-point). *M*: MT-stained skin sections (×20 magnification; scale bar: 100 µm) from BLM OA+PUMP (15 + 100) and BLM OA+PUMP (15 + 100+NTD) mice at *day 42*. Variations in dermal (*N*) and hypodermal thickness (*O*) given as mean ± SD (*n* = 5 mice/group) are presented as scatter plots. Data presented in *B–G*, *I*, *L*, *N*, and *O* were analyzed for statistical significance by one-way ANOVA+Dunnett’s test for multiple comparison; *P < 0.05, **P < 0.01, ***P < 0.001 vs. BLM OA+PUMP (15 + 100). Statistical significance of the data in *J* was evaluated by two-way ANOVA+Dunnett’s multiple comparison; *P < 0.05, **P < 0.01, ***P < 0.001 vs. BLM OA+PUMP (15 + 100). BLM, bleomycin; micro-CT, micro-computed tomography; MT, Masson’s trichrome; NTD, Nintedanib; OA, oropharyngeal aspiration.

Representative coronal micro-CT images for the BLM (15 + 100) and Nintedanib (NTD) groups are shown in Fig. 8*A* (see Supplemental Fig. S2 for the saline control). The alterations associated to the mixed-mode BLM treatment (15 + 100) revealed by micro-CT at *day 42* post-OA were partially mitigated by NTD treatment. Specifically, the BLM (15 + 100)-induced decrease in %Normo (Fig. 8*B*) as well as the increase in %Hypo (Fig. 8*C*) and %Non (Fig. 8*D*) were moderately, albeit nonsignificantly, mitigated by NTD. On the other hand, “tissue,” which reflects both ECM deposition and inflammatory infiltration and was significantly augmented by mixed-mode BLM (15 + 100) administration (*P* < 0.001 vs. saline), was practically unaffected by NTD treatment (Fig. 8*E*). More prominent effects were observed in NTD-treated mice with regard to %Gas, in both expiratory (Fig. 8*F*) and inspiratory (Fig. 8*G*) phases, with a significant increase of the latter parameter (*P* < 0.05) compared with the untreated, BLM (15 + 100) group.

Comparative lung histological analyses were also performed, and representative lung sections from untreated BLM (15 + 100) and NTD-treated mice stained at *day 42* with MT are shown in Fig. 8*H* (see Supplemental Fig. S2 for a representative image of the saline group). Also, although no significant NTD-mediated fibrosis modulation was revealed by the Ashcroft score (Fig. 8*I*), a significant reduction in severe fibrosis grades with a concomitant increase in mild grades (*P* < 0.05 vs. untreated BLM 15 + 100) was revealed by the Ashcroft score frequency distribution (Fig. 8*J*). Consistent with Ashcroft score measurements, lung collagen content was not modulated by NTD. This is documented by representative images of PS-stained lung sections (Fig. 8*K*; see Supplemental Fig. S2 for a representative image of the saline group) as well as by collagen quantification data expressed as % of tissue area (Fig. 8*L*). Histomorphometric examination of skin sections (see representative images for the BLM and NTD groups in Fig. 8*M* and for the saline control group in Supplemental Fig. S2) revealed a significant decrease of dermal thickness in NTD-treated mice (*P* < 0.01 vs. BLM 15 + 100; Fig. 8*N*), associated with an appreciable but nonsignificant increase in hypodermis thickness compared with the BLM (15 + 100) group (Fig. 8*O*).

As previously observed (see data reported above), BALF WBCs were found to be significantly increased in the BLM OA + Pump 15 + 100 group (*P* < 0.001 vs. saline controls), but such an increase was only marginally and nonsignificantly alleviated by NTD treatment (Supplemental Fig. S1*F*). A similar trend was observed for macrophages, which slightly decreased upon NTD treatment (Supplemental Fig. S1*G*), whereas a significant reduction in lymphocyte levels (*P* < 0.01 vs. BLM 15 + 100) was found to be associated with NTD administration (Supplemental Fig. S1*H*). No significant difference in neutrophil levels was detected between the saline, BLM (15 + 100), and NTD groups (Supplemental Fig. S1*I*).

## DISCUSSION

In the present study, we provide an in-depth characterization of an alternative mouse model of SSc-ILD, which combines oropharyngeal aspiration and subcutaneous mini-pump delivery of BLM to achieve a sustained and progressive lung fibrosis for up to 42 days, coupled with persistent skin fibrotic alterations. The first dose-finding study showed that mixed administration of BLM at high dosage (15 + 100) was the best protocol for simultaneously inducing a progressive pulmonary fibrosis lasting till *day 42* and significant skin changes, as revealed by the combination of pulmonary micro-CT and histological analyses. The second longitudinal study, also based on micro-CT and histological analyses, revealed a sustained pulmonary fibrosis progressing from *day 28* to *day 42*, accompanied by a reduction of skin hypodermal thickness. We also found an increased recruitment of white blood cells, especially T cells and macrophages, in the lungs and BALF, whereas only a slight modulation of these cell populations was detected in the skin by immunofluorescence.

Previous studies aimed at replicating the hallmarks of human SSc-ILD by inducing lung and skin fibrosis in mice have met with only partial success (7, 8, 11, 14). In fact, BLM delivery only relying on osmotic minipumps resulted in mild and spatially limited fibrotic lesions in the lungs, whose intensity gradually decreased after *day 28* (11).

Even though SSc-ILD is more prevalent in female than male animals (23), it has been reported that male mice are more susceptible to BLM-induced lung fibrosis (24, 25). Consistent with these observations, we had to terminate at *day 21* a pilot-study conducted on five male mice, because of excessive severity, body weight loss, and increased aggressiveness (data not shown), all well above the threshold of Humane end point criteria (HEP). This motivated our decision of using female C57Bl/6 mice, which are less aggressive and easier to handle during surgical procedures for pump implantation and removal, as test animals for validating the present model of mixed-mode BLM administration. Despite a significant body weight loss also in female mice, it consistently remained under 20% relative to *day 0*, in accordance with our animal welfare regulations. We started with a dose-finding study to identify the optimal fibrosis induction protocol. We found that the sole administration of BLM via OA (10 + 0 group) induced only limited histomorphometric alterations of the lung parenchyma, such as a slight increase of the Ashcroft score, and did not sustain fibrosis progression. These findings are consistent with those of a previous study that documented the resolution of BLM-induced fibrosis after 28 days (10). In contrast, animals were first treated with BLM via OA followed by osmotic mini-pump, subcutaneous BLM administration (10 + 80, 10 + 100, and 15 + 100 groups) displayed substantial alterations of both pulmonary micro-CT and histo-morphometric readouts, indicating a sustained fibrosis up to 42 days post-OA. In fact, even at this late time, significant alterations of micro-CT-derived parameters (e.g., %Hypo) were observed. Notably, other micro-CT-derived parameters, such as %Normo, %Gas_P01_, and %Gas_P02_, were significantly affected only in the high-dosage (10 + 100 and 15 + 100) groups. These graded responses suggest the development of a progressive and dose-dependent fibrosis triggered by mixed-mode BLM administration. Furthermore, although all BLM dosages delivered via OA + pump resulted in an increased Ashcroft score, only the 10 + 100 and 15 + 100 doses had a significant impact on severe-grade fibrosis.

A similar superiority of the high dosage regimens was also observed at the level BALF inflammatory cells (especially, macrophages and lymphocytes), which were significantly increased at all BLM doses administered with OA + Pump but reached a maximum at the 15 + 100 dose. Dose-dependent fibrotic responses of increasing severity were observed as we progressed from the 10 + 80 to the 15 + 100 dosage, but we refrained from testing even higher BLM doses for animal welfare reasons.

Interestingly, the 10 + 100 and 15 + 100 groups, despite the different amounts of OA-administered BLM, displayed similarly compromised pulmonary parameters at 42 days, suggesting that pump-delivered BLM plays a somewhat dominant profibrotic role at least at the latest time-point. This interpretation is supported by the distinctive body weight trends observed in the different animal groups. In fact, although significant body-weight losses were initially apparent in all groups, the 10 + 80 group displayed a clear trend toward body-weight normalization after *day 21*, whereas a further body-weight decline was observed in the 10 + 100 and 15 + 100 groups.

We hypothesize that the initial weight loss may be attributed to the effects of the surgical procedure for pump implantation, in addition to the impact of OA-delivered BLM. Subsequent weight losses, instead, are likely associated with BLM release from the pumps and fibrosis establishment. A similar trend in body weight loss was observed in our previous studies on the BLM subcutaneous minipump model (11). However, at variance with the present mixed-mode administration model, the lungs of mice only subjected to minipump BLM administration displayed a fibrosis restricted to the subpleural area and pulmonary lesions tended to spontaneously resolve after 21 days post-pump implantation. It is thus difficult to directly compare these previous results with those obtained using the mixed-mode BLM administration model, where the examination of later time-points (i.e., *day 28*, *35*, and *42*) revealed the presence of persistent and severe fibrotic alterations. Regarding skin parameters, only the 15 + 100 group showed significant skin alterations at *day 42*: namely, a significant decrease in hypodermis thickness, while dermal thickness was only moderately affected. The reduction in hypodermis thickness may stem from an intensified fibrotic response triggered by the highest BLM dose, leading to a significant hypodermal restructuring. Conversely, the moderate change in dermal thickness suggests potential variations in dermal sensitivity to the fibrotic effects brought about by bleomycin a hypothesis that warrants further investigation in future studies.

Based on the results of the dose-finding study, we conducted a time-course analysis with the 15 + 100 dose and investigated fibrosis progression at three late time-points (i.e., *day 28*, *35*, and *42*). Micro-CT lung imaging at *day 7* provided a baseline profile before pump implantation. Although micro-CT imaging is noninvasive, it requires full anesthesia. We thus decided not to perform micro-CT scans on *days 14* and *21*, a time-window coinciding with the peak of body weight loss, to avoid any possible side-effect on, and artifactual exacerbation of, animal welfare. Micro-CT data confirmed a progressive fibrotic deterioration in mice treated with BLM 15 + 100, with increasing %Hypo and %Non as well as tissue, and a decrease of % gas in both the inspiratory (P01) and the expiratory (P02) phases. In keeping with micro-CT data, histological analysis indicated a sustained lung fibrosis with significant extracellular matrix deposition persisting up to *day 42*. The Ashcroft score and its frequency distribution highlighted an escalation in severe fibrosis at *day 42*, with a prominence of severe-grade fields consistently observed throughout the time-course study. Fibrosis persistently increasing up to *day 42*, as revealed by both micro-CT and histological data, is the major distinguishing feature of the present mixed-mode BLM administration model compared with previous models, in which BLM was only administered subcutaneously by osmotic mini-pump delivery (11, 14). In contrast to the peak of fibrosis at *day 42* indicated by the Ashcroft score, collagen staining of PS-stained sections showed a higher collagen accumulation at *day 35*. This discrepancy may reflect the multifaceted nature of the overall fibrotic process, which is not limited to collagen synthesis and maturation but also includes inflammatory and immune response components (26, 27). Given this kind of complexity, it is reasonable to imagine that the combination of different bioanalytical approaches can provide a more comprehensive representation of the dynamic changes taking place during fibrosis progression.

The high-dosage study also confirmed a marked reduction of the hypodermal layer persisting up to *day 42*, accompanied by a slight increase in dermal thickness at the same time-point.

Immunofluorescence analysis revealed an increase of both Col 1a1 and biglycan in the 15 + 100 BLM group. Here, again, there was a difference between collagen levels detected with the use of Picrosirius staining or collagen 1a1 immunofluorescence, which can be attributed to the inherent specificities of the two analytical techniques and the complex nature of collagen composition and remodeling in fibrotic tissues (26–28). The observed rise in lung levels of biglycan upon mixed-mode BLM administration likely reflects an accumulation of extracellular matrix (29) associated to scarring and tissue damage. The more sustained increase in biglycan compared with collagen 1a1 points to a pivotal role of this extracellular matrix component in the development of fibrotic alterations and further suggests a composite response to the complex microenvironmental changes associated with fibrosis establishment. On the other hand, the skin biglycan immunostaining signal was below the quantification threshold, suggesting a limited physiological role of this proteoglycan in the dermis, as also noted in a previous study (30).

The development of fibrotic alterations also entails an augmented recruitment of immune cells, particularly T cells and macrophages, which through the release of profibrotic cytokines and autoantibodies play a crucial role in promoting fibrosis progression and inflammatory vasculopathies, a notable feature of SSc-ILD. While the present work was primarily focused on fibrosis, future studies specifically addressing blood vessel inflammation biomarkers will be essential to gain a broader understanding of the BLM mixed-mode administration model.

Macrophages and T cells markedly increased throughout the entire time-course both in the lungs and in BALF. Their peak at *day 35* may point to a critical phase of the inflammatory process, potentially linked to the subsequent peak of fibrotic alterations revealed at *day 42* by both micro-CT and histological analyses (31, 32).

Despite the lack of significant variations in skin-related inflammatory cells, we observed a slight modulation of T cells levels by immunofluorescence, whose recruitment increased over time, as well as macrophages, which are thought to be involved in early stages of systemic sclerosis (SSc) progression (11, 31, 32). In fact, an appreciable skin inflammation, somewhat attenuated at *day 42* compared with earlier time-points, was revealed by histomorphological analyses. Notably, the lower modulation of skin immune cells compared with pulmonary WBCs observed at *day 35*, was mirrored by fibrotic skin alterations of lower intensity at *day 42*. A future investigation of the specific T cell subtypes involved, and the activation state of macrophages might provide more detailed insights into the nature of this immune response and its contribution to fibrosis development.

We also determined the levels of PPAR-γ, a transcriptional regulator of metabolic processes that promote adipogenesis while inhibiting collagen deposition (33). Reduced PPAR-γ levels have previously been reported in patients with SSc (33, 34) as well as after BLM administration in a mouse model of SSC (35). In this study, the decrease of skin PPAR-γ levels after BLM OA + Pump 15 + 100 treatment was found to correlate with weight loss and increased dermal collagen, thus pointing to this transcription factor as a potentially useful biomarker and a therapeutic target worth of further investigation.

To further explore the relevance of the mixed-mode BLM administration model and preliminarily test its applicability for drug discovery, a subset of mice subjected to the BLM (15 + 100) regimen were treated daily with Nintedanib starting on *day 28* and examined by micro-CT and histo-morphometric analyses on *day 42*. This particular time-window was chosen because of its concurrence with the stabilization of fibrotic lesion intensity and body weight variation as reveled by the dose-finding study.

At 42 days post-OA, only a moderate mitigation of the alterations induced by mixed-mode BLM delivery was observed in the NTD-treated mice.

This included a significant increase in %Gas, a lowering of severe-grade fibrosis, and a reduced accumulation of lymphocytes in BALF, in line with previous findings in the BLM model (36). We speculate that this limited efficacy might be due to a suboptimal time-window for pharmacological intervention. In fact, NTD administration was started when fibrotic lesions were already well detectable and practically irreversible. On the other hand, an earlier treatment initiation would have overlapped with the phase of acute weight loss, further increasing the stress due to daily manipulations. This represents an inherent limitation of the mixed BLM delivery model, that will require further optimization studies, including the setting-up of alternative dosing schedules better suited to pharmacological treatment with antifibrotic and anti-inflammatory drugs as well as novel candidate compounds.

Overall, we believe that the approach presented in this study represents a relevant advancement in the field of SSc-ILD preclinical models. Indeed, although the BLM model only relying on the use of osmotic pumps proved to be less stressful for the animals and resulted in a more homogeneous fibrosis development compared with that produced by multiple subcutaneous BLM injections (11), mixed-mode BLM administration overcomes the spatial and temporal limitations of pulmonary fibrotic lesions. The extended lung fibrotic response and concomitant involvement of the hypodermal layer induced by our mixed-mode of BLM administration provides a unique opportunity to explore specific pathogenetic mechanisms and potential therapeutic targets for SSc-ILD.

Unfortunately, a marked weight loss, albeit compliant with current animal welfare standards, precluded the testing of even higher BLM dosages, thus limiting the extent of the observed dermal alterations, and hindered the use of male mice, thereby limiting the clinical translatability of this model. Moreover, further investigations will be required to determine an optimal timing for antifibrotic treatment. Despite these limitations, our study represents a valuable alternative mouse model of pulmonary and cutaneous fibrosis, that holds important implications for the development of novel therapeutic interventions as well as for a more detailed understanding of the cellular and molecular mechanisms underlying the pathogenesis of fibrotic diseases.

## DATA AVAILABILITY

The datasets generated, utilized and/or analyzed in the current study are available from the corresponding author on a reasonable request.

## SUPPLEMENTAL DATA

10.6084/m9.figshare.24886059 Supplemental Fig. S1: https://doi.org/10.6084/m9.figshare.24886059.
Supplemental Fig. S2: https://doi.org/10.6084/m9.figshare.24886185.

## GRANTS

This study was fully supported by Chiesi Farmaceutici S.p.A. and by the National Plan for NRRP Complementary Investments (PNC, established with the decree-law 6 May 2021, no. 59, converted by law no. 101 of 2021) in the call for the funding of research initiatives for technologies and innovative trajectories in the health and care sectors (Directorial Decree no. 931 of 06-06-2022)—project no. PNC0000003—AdvaNced Technologies for Human-centrEd Medicine (project acronym: ANTHEM).

## DISCLAIMERS

This work reflects only the authors’ views and opinions, neither the Ministry for University and Research nor the European Commission can be considered responsible for them.

## DISCLOSURES

This study was fully supported by Chiesi Farmaceutici S.p.A. A.G., G.V., and F.F.S. are employees of Chiesi Farmaceutici S.p.A., which supported the research work. None of the other authors has any conflicts of interest, financial or otherwise, to disclose.

## AUTHOR CONTRIBUTIONS

A.G., E.F., and F.F.S. conceived and designed research; A.G., E.F., M.Z., and Z.K. performed experiments; M.Z. and D.B. analyzed data; A.G., E.F., F.P., R.C., and F.F.S. interpreted results of experiments; A.G., E.F., M.Z., D.B., and R.C. prepared figures; A.G. and F.F.S. drafted manuscript; E.F., F.P., and G.V. edited and revised manuscript; A.G., E.F., M.Z., D.B., F.P., Z.K., R.C., G.V., and F.F.S. approved final version of manuscript.
